# Repeatability of tumor blood flow quantification with ^82^Rubidium PET/CT in prostate cancer — a test-retest study

**DOI:** 10.1186/s13550-019-0529-2

**Published:** 2019-07-04

**Authors:** Mads Ryø Jochumsen, Kirsten Bouchelouche, Katrine Bødkergaard Nielsen, Jørgen Frøkiær, Michael Borre, Jens Sörensen, Lars Poulsen Tolbod

**Affiliations:** 1 Department of Nuclear Medicine and PET-Centre, Aarhus University Hospital, Palle Juul-Jensens Boulevard 165, 8200 Aarhus N, Denmark; 2 Department of Clinical Medicine, Aarhus University, Aarhus, Denmark; 3 Department of Public Health, Section for Biostatistics, Aarhus University, Aarhus, Denmark; 4 Department of Urology, Aarhus University Hospital, Aarhus, Denmark

**Keywords:** Tumor blood flow, Prostate cancer, ^82^Rubidium-PET, Test-retest, Repeatability

## Abstract

**Background:**

Non-invasive tumor blood flow (TBF) quantification is a candidate approach for risk stratification and monitoring of prostate cancer patients. Validation data have recently been published on prostate TBF measurement with the widely used positron emission tomography (PET) flow tracer ^82^Rubidium (^82^Rb). However, no test-retest data is available for TBF measurement with ^82^Rb PET in prostate cancer. Such information is important to determine the potential clinical usefulness of the technique. The aim of the present study was to determine the test-retest repeatability of TBF measurement with both dynamic and static ^82^Rb PET.

**Methods:**

We recruited 10 low-to-high-risk prostate cancer patients scheduled for clinical prostate-specific membrane antigen (PSMA) PET/computed tomography (CT) or magnetic resonance imaging. Pelvic and cardiac static and dynamic ^82^Rb PET/CT were performed at baseline and repeated on a different day within 1 week. In total, 11 primary lesions were analyzed.

**Results:**

For K1, standardized uptake values (SUV)max, SUVmean, and SUVpeak, prostate cancer ^82^Rb PET TBF has a repeatability of 32%, 51%, 53%, and 58% and an intraclass correlation of 0.98, 0.89, 0.88, and 0.88, respectively.

**Conclusion:**

Dynamic ^82^Rb PET/CT with kinetic modeling measures TBF in prostate cancer with high repeatability, which allows identification of blood flow changes of 32%. Static late-uptake ^82^Rb PET/CT is inferior, and only intra-individual blood flow changes above 51% can hence be recognized.

## Background

One of the principal hallmarks of cancer is angiogenesis [1], which leads to increased blood flow in the tumor. Tumor blood flow (TBF) imaging has therefore previously been studied for characterization and treatment response evaluation in various cancers, including non-small cell lung cancer [2, 3], breast cancer [4–7], head and neck cancer [8], colorectal cancer [3, 9], brain cancer [10], and prostate cancer [11–13]. ^15^O-H_2_O positron emission tomography (PET) is the gold standard method for non-invasive blood flow imaging. Several studies have found a high reproducibility of TBF measurements with ^15^O-H_2_O PET in various cancers [14–17]. Clinical implementation of ^15^O-H_2_O PET is, however, challenging and limited to few PET centers due to the requirement of an on-site cyclotron to produce the short-lived ^15^O tracer. An alternative flow tracer is the generator-produced potassium analogue ^82^Rubidium (^82^Rb), which is already clinically available for cardiac blood flow measurement at many PET centers worldwide. ^82^Rb is a retention tracer that allows both kinetic modeling and static analysis of late uptake images using standardized uptake values (SUV). Enhanced ^82^Rb uptake has previously been described in breast cancer [18], lung cancer, lymphoma, multiple myeloma [19], malignant pheochromocytoma [20], and metastatic renal cell carcinoma [21]. We have recently demonstrated three novel insights: first, that TBF measures derived from static and dynamic ^82^Rb PET were highly correlated with ^15^O-H_2_O PET; second, that ^82^Rb TBF in prostate cancer was significantly larger than blood flow in healthy prostate tissue; and third, that ^82^Rb TBF correlates with prostate cancer aggressiveness [22].

The clinical usefulness of TBF measurement with ^82^Rb PET is highly dependent on the repeatability of the method. To design and interpret active surveillance and treatment response studies, knowledge of the test-retest variability is also required. ^82^Rb PET has a high reproducibility in quantitative myocardial blood flow assessment [23]. However, no test-retest data on ^82^Rb PET on tumors exist. Hence, the aim of the present study was to evaluate the repeatability of TBF measurement with static and dynamic ^82^Rb PET.

## Methods

### Patient population

To evaluate the repeatability of TBF measurement with static and dynamic ^82^Rb PET, ten patients diagnosed with prostate cancer were included in the study. Both low-risk patients in active surveillance and high-risk patients were recruited as we aimed to represent tumors spanning the range from low, intermediate, to high blood flow. The low-risk patients had undergone a clinical multiparametric magnetic resonance imaging (MRI) scan, and the high-risk patients had undergone a clinical ^68^Ga-prostate-specific membrane antigen (PSMA) PET/computed tomography (CT). Each patient underwent two ^82^Rb PET scan sessions within 1 week, each consisting of a dynamic pelvic and a dynamic cardiac ^82^Rb PET. No patients were excluded from the study.

### Imaging

All ^82^Rb PET scans were carried out on a GE Discovery MI Digital Ready PET/CT (GE Healthcare, Waukesha, WI, USA). At the beginning of each scan, a bolus of 1110 MBq ^82^RbCl was injected directly by the Cardiogen-82 generator infusion system (Bracco, Monroe Township, NJ, USA). Details of the scan and reconstruction protocols have been described previously [22].

### Image analysis

#### Static analysis

**Fig. 1:**
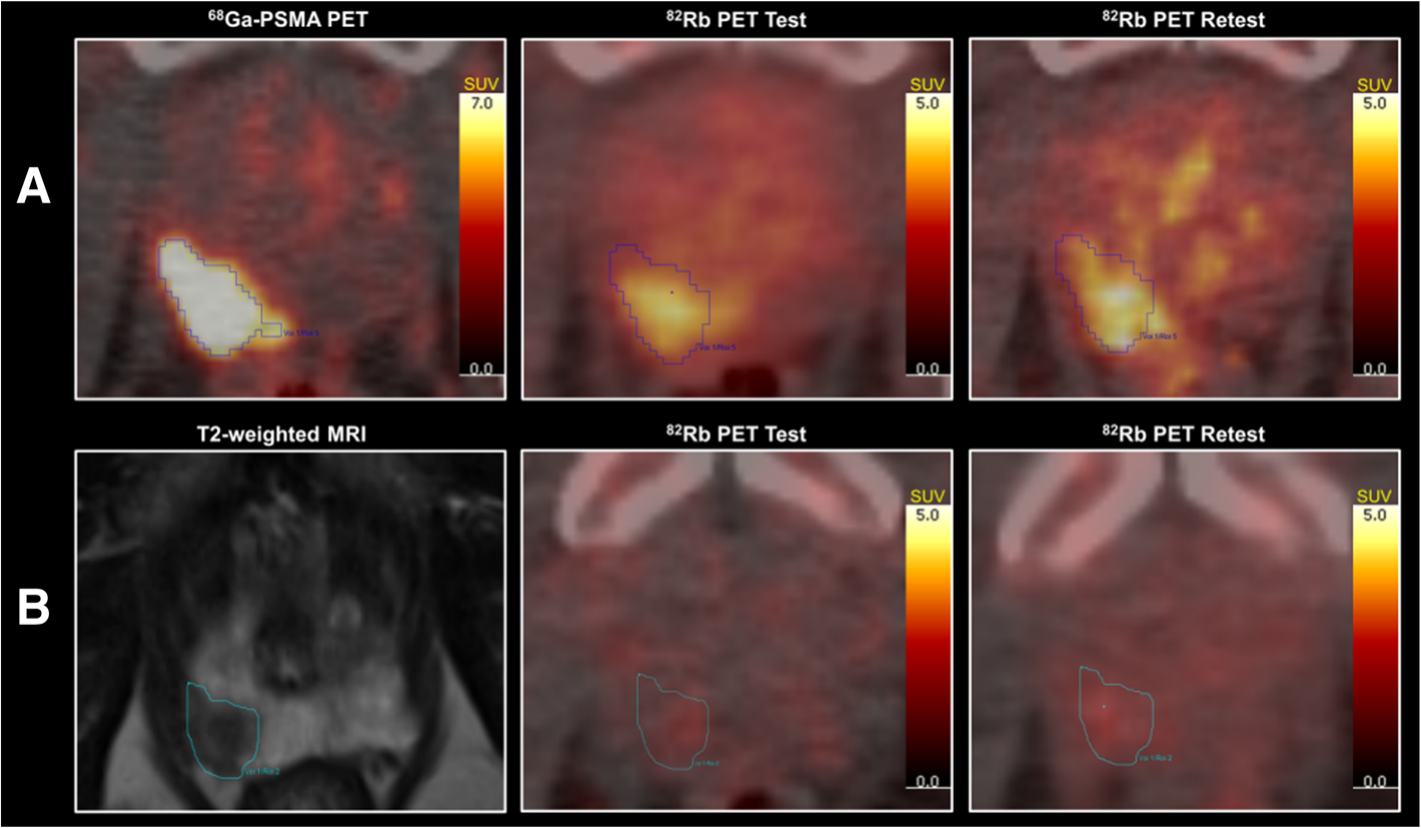
Images of two patients in the study. **a** Patient 2, a high-risk patient with a Gleason 5+4 high-flow tumor and metastatic disease. To the left, ^68^Ga-prostate-specific membrane antigen (PSMA) positron emission tomography (PET)/computed tomography (CT) with automatically drawn volumes of interest (VOIs) from standard uptake value (SUV) 6 fixed threshold in blue. The first ^82^Rb PET/CT scan in the middle and the second scan to the right. **b** Patient 9, a low-risk patient in active surveillance with a low-flow tumor. To the left, t2-weighted magnetic resonance imaging with hand-drawn VOIs in light blue. The first ^82^Rb PET/CT scan in the middle and the second scan to the right

In seven patients, where ^68^Ga-PSMA PET/CT scans were available, the ^68^Ga-PSMA PET/CT scans were co-registered to the two ^82^Rb PET/CT scans using the low-dose CTs (Hybrid Viewer, Hermes Medical Solutions, Stockholm, Sweden). The tumor volumes of interest (VOIs) were drawn directly on the ^68^Ga-PSMA PET/CT images and subsequently transferred to the ^82^Rb PET/CT static images. The tumor VOIs were automatically drawn in two ways, with a fixed SUV threshold of 6 and by using a 30% threshold of max at the tumor site. An example of a high-risk patient with PSMA SUV 6 fixed threshold VOIs is shown in Fig. 1a with corresponding test and retest ^82^Rb PET/CT scans. Multiple different SUV values and different threshold percentages were evaluated before selecting SUV 6 and 30% as the most optimal threshold. One patient had a tumor in the prostate basis, the PSMA activity of which was confluent with the urinary PSMA activity. Consequently, it was necessary to mask the bladder manually before the automatic VOI drawing.

In the remaining three patients, T2-weighted images of the MRI scans were co-registered to the ^82^Rb PET/CT scans using the low-dose CT as a bridge. The tumor VOIs were drawn directly on the MRI by visual guidance. An example of a low-risk patient with MRI-guided VOIs is shown in Fig. 1b, including both ^82^Rb PET/CT scans of the patient.

The static image series (3 to 7 min post injection) were used for SUV analysis. Image analysis was performed using Hermes viewer (Hermes Medical Solutions, Stockholm, Sweden).

#### Dynamic analysis

The tumor VOIs described above were transferred to the dynamic PET series, and time-activity curves were extracted. To obtain a blood input function for calculation of K1, we utilized the method developed by Tolbod et al. [13], which uses an image-derived input function from a separated scan of the heart. In short, a separate ^82^Rb PET/CT scan over the heart was performed in connection with the pelvic scan with the same tracer dose and infusion profile. Image-derived input functions were extracted with cluster analysis from both the heart and pelvic scans, and subsequently, the heart image-derived input function was delay- and dispersion-corrected to the pelvic image-derived input function. This method was previously validated against ^15^O-H_2_O PET with input function obtained using arterial blood sampling [13]. Kinetic modeling was performed using a one-tissue compartment model.

### Statistical analysis

Since the clinically relevant parameter is a relative change in blood flow, the repeatability data for both K1, SUVmax, SUVmean, and SUVpeak were log-transformed. The data were visually inspected for normality using Q-Q plots of the differences, and based on Bland-Altman plots, the variation does not seem to depend on the average [24]. The repeatability of the method was calculated by the method described by Bland and Altman [25]. The within-patient/within-lesion coefficient of variance, repeatability, and intraclass coefficients (ICCs) were calculated for both K1, SUVmax, SUVmean, and SUVpeak. The statistical parameters and formulas used are described in details in Lodge et al. [15]. Bland-Altman plots are presented in the original scale with back-transformed limits of agreement using the methodology of Euser et al. [26]. Sample size calculations were performed to detect relative changes of − 20%, − 30%, and − 50% using a two-sided significance test of no difference for paired log-normally distributed data with a significance level of 5% and a power of 95%. For these sample size calculations, the standard deviation for the difference between the logarithm of the test and the logarithm of the retest was used.

Study data were collected and managed using REDCap (Vanderbilt University Medical Center, Nashville, TN, USA) electronic data capture tools, hosted at Aarhus University [27]. Data analysis was performed using Stata version 15.1 (StataCorp LLC, College Station, TX, USA).

## Results

The dynamic and static TBF measurements of test and retest scans are shown in Table 1 for VOIs drawn from PSMA SUV 6 fixed threshold. In total, 11 primary lesions were analyzed, as patient 8 had two PIRADS 4 lesions on MRI.

**Table 1:** Tumor blood flow values for both test and retest scans of all patients for volumes of interest (VOIs) drawn from standardized uptake value (SUV) 6 fixed threshold on prostate-specific membrane antigen (PSMA) positron emission tomography (PET)/computed tomography (CT)

Patient ID	Tumor size (cm^3^)	Test				Retest			
		K1	SUVmax	SUVmean	SUVpeak	K1	SUVmax	SUVmean	SUVpeak
1	3.92	0.36	5.00	2.99	3.88	0.34	5.14	3.20	3.84
2	21.30	0.27	4.10	2.55	3.58	0.25	5.42	3.10	4.18
3	0.46	0.05*	1.44*	1.12*	1.22*	0.05*	1.50*	1.15*	1.11*
4	2.21	0.12	3.77	2.52	2.77	0.10	5.27	3.38	3.31
5	13.49	0.22	5.50	2.46	4.45	0.16	6.26	3.09	5.15
6	36.83	0.23	6.04	3.61	4.96	0.29	4.85	2.93	3.86
7	0.88	0.23	4.97	3.73	3.77	0.26	4.85	4.02	4.09
8	0.84	0.08*	3.15*	1.57*	2.16*	0.09*	2.25*	1.05*	1.33*
	0.91	0.13*	2.62*	1.57*	2.36*	0.12*	2.07*	1.18*	1.48*
9	1.47	0.05*	1.63*	0.93*	1.16*	0.05*	1.67*	1.11*	1.38*
10	18.99	0.43	5.99	3.02	5.05	0.40	4.06	2.19	3.32

^*^VOIs are drawn from magnetic resonance imaging

Full scan analysis with VOI drawing using 30% threshold on the PSMA PET was also performed. There was no noteworthy difference between the TBF measurements and the resulting repeatability based on the VOI drawing methods. Therefore, the results from the 30% threshold VOI have been left entirely out of the paper for simplicity.

The descriptive test-retest statistics are found in Table 2.

**Table 2:** Descriptive test-retest statistics. *ICC* intraclass coefficient

Measure	ICC	Repeatability (%)
K1	0.98	32
SUVmax	0.89	51
SUVmean	0.88	53
SUVpeak	0.88	58

**Fig. 2:**
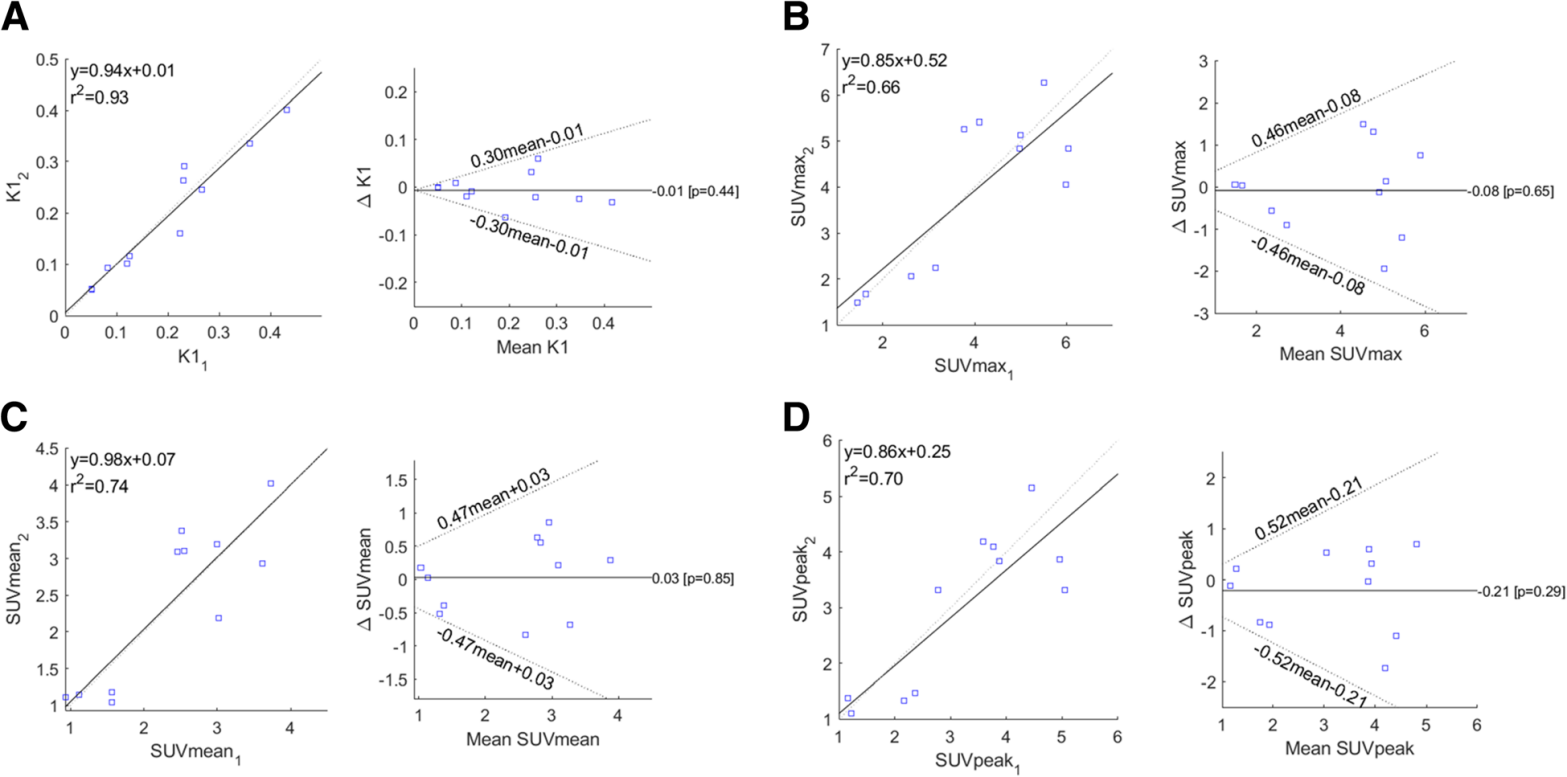
Replicate measures are plotted against each other for K1 (**a**), SUVmax (**b**), SUVmean (**c**), and SUVpeak (**d**). Gray dashed line represents *y* = *x*, whereas the solid black line is the linear fit. Linear equation and Pearson’s *r*^2^ are shown. Bland-Altman plots of back-transformed data for K1 (**a**), SUVmax (**b**), SUVmean (**c**), and SUVpeak (**d**). The black solid line is the mean difference between measurement 2 and 1. Black dotted lines are 95% upper and lower 95% limits of agreement (1.96 × sd on the log-transformed data)

The data from the test and retest scans have been plotted against each other, and linear regression analysis has been performed for both K1 (Fig. 2a), SUVmax (Fig. 2b), SUVmean (Fig. 2c), and SUVpeak (Fig. 2d). Similarly, Bland-Altman plots of the back-transformed data are shown for all measured parameters with 95% upper and lower limits of agreement shown (Fig. 2a–d).

Sample size implications of the data from the present study can be found in Table 3.

**Table 3:** Sample size implications of tumor blood flow repeatability from the present study. Calculations were performed to detect relative changes of − 20%, − 30%, and − 50% using a two-sided significance test for paired data with a significance level of 5% and a power of 95%. Log-transformed data were used for all calculations

	K1	SUVmax	SUVmean	SUVpeak
Sample size (*N*) at − 20% change	9	17	18	21
Sample size (*N*) at − 30% change	5	8	9	10
Sample size (*N*) at − 50% change	3	4	5	5

**Fig. 3:**
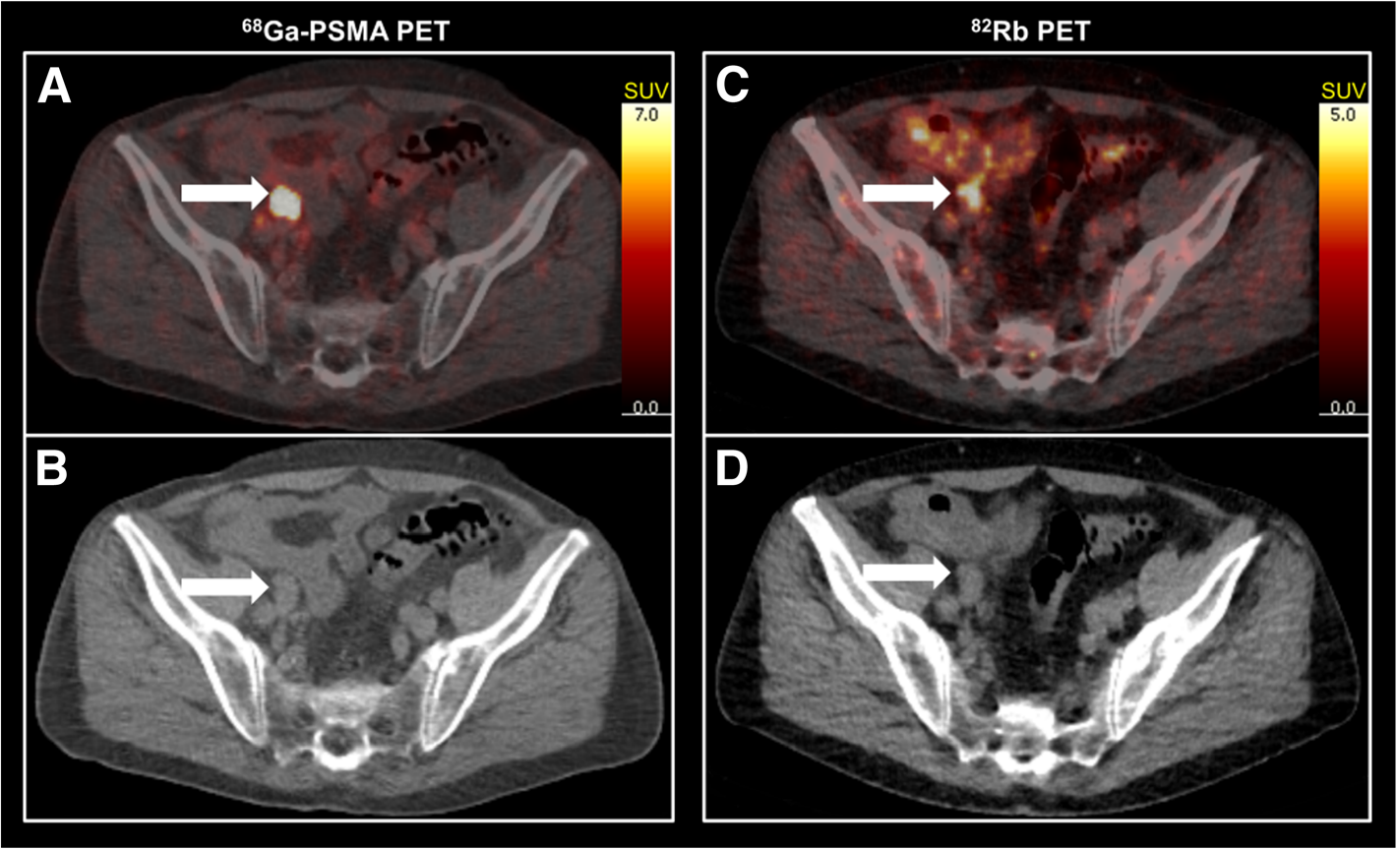
Images of a pelvic lymph node metastasis (patient 2). To the left, ^68^Ga-prostate-specific membrane antigen (PSMA) positron emission tomography (PET)/computed tomography (CT)-fused image (**a**) (SUVmax 43.0) and corresponding low-dose CT (**b**). To the right, ^82^Rb PET/CT fused image (**c**) (SUVmax 6.5) and corresponding low-dose CT (**d**)

One patient (patient 2) had lymph node metastases, which also displayed markedly elevated blood flow compared with surrounding soft tissues (Fig. 3). The metastases were not assessed in the repeatability analysis.

## Discussion

The main findings of the present study are that the repeatability of TBF measurement was approximately 32% for dynamic ^82^Rb PET/CT and 51–58% for static ^82^Rb PET/CT depending on the SUV measure used.

With 10 patients and 11 primary lesions, we are in line with the two small retest studies on ^15^O-H_2_O PET on tumors [16, 28], but our study is considerably smaller than the large study by Lodge et al. [15]. A further limitation of the present study is that the tumor VOIs were drawn by two different methods. The tumor VOIs were drawn on PSMA PET/CT using objective parameters, which makes the VOIs reproducible. By contrast, the VOIs on MRI were drawn by hand according to the best assessment of the tumor’s delineation. The fusion of the scans was performed with utmost thoroughness, but this approach inevitably introduces an element of uncertainty as the patients do not lie in the exact same position in each scan.

By scanning the patients on different days, we include the day-to-day variability in our data. The limitation of the present study design is that we do not assess the exact repeatability of the method itself, but a mixture of physiological variation and scan repeatability. The strength of the design on the other hand is that it mimics a clinical setting. In the comprehensive work by Lodge et al. [15], the authors discuss the likely underestimation of the repeatability in their study design due to same-day scanning of the patients and the absence of moving between scans. The same regards for Van der Veldt et al. [16], which has a similar study design. De Langen et al. [28] scanned the patients on different days, but added a 60-min ^18^F-fluorothymidine scan each day for more precise VOI definition. Because of the abovementioned factors, a larger variability must be expected in the present study than in previous studies.

### Dynamic tumor blood flow measurement

The repeatability of ^82^Rb PET K1 was approximately 32% in the present study, which means that the absolute difference of two measurements of the same tumor relative to the mean is expected to be below 32% for 95% of the pairs of observations. In other words, an absolute difference of more than 32% is expected to be an actual change in TBF due to progression or treatment effect. The reproducibility of dynamic ^82^Rb PET TBF was comparable to the results of the test-retest studies on ^15^O-H_2_O PET, which have found a repeatability of 16% [16], 18% [28], and 37% [15]. Furthermore, the reliability of ^82^Rb PET K1 was excellent with an ICC of 0.98 [29]. A high ICC demonstrates that the intra-tumor variation is small compared to the inter-tumor variation.

### Static tumor blood flow measurement

The repeatability for the static SUV measures of ^82^Rb PET are decidedly inferior to those of dynamic ^82^Rb PET K1 and, hence, also to ^15^O-H_2_O PET K1 [15, 16, 28]. Using the same arguments as above, static ^82^Rb PET is able to detect changes above 51% in TBF in repeated measurements. The repeatability was numerically better for SUVmax than for SUVmean and poorest for SUVpeak (Table 2). The ICCs were equally good for all static measures [29]. The difference between SUVmax and SUVmean is large for the largest tumors, which is probably explained by the heterogeneity of the tumor perfusion. The study population is, however, too small to make conclusions on this.

Due to the short half-life of ^82^Rb and the fast nature of its uptake and wash-out kinetics, SUV calculated from late uptake images suffer from lower count statistics compared to the first part of the uptake curve and is sensitive to even small timing inconsistencies and differences in the infusion profile of the tracer. In this light, it is reasonable that static SUV measures exhibit a lower repeatability compared to dynamic K1.

### Future perspectives

The repeatability of ^82^Rb PET in the present study would be acceptable for assessing the TBF state (high TBF, moderate TBF, and low TBF) as a rough risk stratification in combination with MRI. Also, with the reported threshold of a 32% change, dynamic ^82^Rb PET may be able to detect relevant blood flow changes as response to prostate cancer treatment. These applications need to be investigated in further studies.

Since low-risk prostate cancer is slow growing, a 32% change in blood flow is relatively large for this population. Hence, it is doubtful that ^82^Rb PET can be used for precise monitoring of low-risk prostate cancer patients in active surveillance.

Based on results from the present study, dynamic ^82^Rb PET should be preferred to static ^82^Rb PET in future studies due to its markedly higher repeatability. Dynamic ^82^Rb PET K1 could be precise and consistent enough to draw conclusions on an individual patient basis, whereas static ^82^Rb PET with SUV measures is probably more suited for drawing conclusions on a population basis. However, the disadvantages of dynamic ^82^Rb PET are the more cumbersome image analysis requiring specialized kinetic modeling software as well as the extra cost and radiation dose (approximately 1 mSv) from the supplementary ^82^Rb PET heart scan. Static ^82^Rb PET remains a pragmatic alternative if the expected treatment response is thought to be larger than 50%, due to the simple acquisition and image analysis. Sample size calculations can be found in Table 3. It appears that SUV is an excellent measure for studies with matched pairs.

## Conclusions

TBF measurement with dynamic ^82^Rb PET K1 is repeatable in prostate cancer. When monitoring patients repeatedly, a change of more than 32% is likely to be an actual change in the TBF. Static ^82^Rb PET/CT for TBF measurement has a repeatability above 51%, but may still be relevant on a population basis due to its simple image analysis.

## Data Availability

The dataset supporting the conclusions of this article is included within the article.

## Abbreviations

^82^Rb: ^82^Rubidium
CT: Computed tomography
MRI: Magnetic resonance imaging
PET: Positron emission tomography
PSMA: Prostate-specific membrane antigen
SUV: Standardized uptake values
TBF: Tumor blood flow
VOIs: Volumes of interest
